# Energy system contribution in a maximal incremental test: correlations with pacing and overall performance in a 10-km running trial

**DOI:** 10.1590/1414-431X20154787

**Published:** 2015-09-18

**Authors:** M.V. Damasceno, L.A. Pasqua, A.E. Lima-Silva, R. Bertuzzi

**Affiliations:** 1 Grupo de Estudos em Desempenho Aeróbio da USP (GEDAE-USP), Departamento de Esporte, Escola de Educação Física e Esporte, Universidade de São Paulo, São Paulo, SP, Brasil; 2 Grupo de Pesquisa sobre Ciência dos Esportes, Centro Acadêmico de Vitória, Universidade Federal de Pernambuco, Vitória de Santo Antão, PE, Brasil

**Keywords:** Aerobic metabolism, Running, Maximal oxygen uptake, Anaerobic metabolism, Endurance performance

## Abstract

This study aimed to verify the association between the contribution of energy systems during an incremental exercise test (IET), pacing, and performance during a 10-km running time trial. Thirteen male recreational runners completed an incremental exercise test on a treadmill to determine the respiratory compensation point (RCP), maximal oxygen uptake (${\dot{\text{V}}\text{O}}_{2\max}$), peak treadmill speed (PTS), and energy systems contribution; and a 10-km running time trial (T10-km) to determine endurance performance. The fractions of the aerobic (W_AER_) and glycolytic (W_GLYCOL_) contributions were calculated for each stage based on the oxygen uptake and the oxygen energy equivalents derived by blood lactate accumulation, respectively. Total metabolic demand (W_TOTAL_) was the sum of these two energy systems. Endurance performance during the T10-km was moderately correlated with RCP, ${\dot{\text{V}}\text{O}}_{2\max}$and PTS (P<@0.05), and moderate-to-highly correlated with W_AER_, W_GLYCOL_, and W_TOTAL_ (P<0.05). In addition, W_AER_, W_GLYCOL_, and W_TOTAL_ were also significantly correlated with running speed in the middle (P<0.01) and final (P<0.01) sections of the T10-km. These findings suggest that the assessment of energy contribution during IET is potentially useful as an alternative variable in the evaluation of endurance runners, especially because of its relationship with specific parts of a long-distance race.

## Introduction

Traditionally, physiological and mechanical variables, such as maximal oxygen uptake (${\dot{\text{V}}\text{O}}_{2\max}$) (1), metabolic thresholds (2), and peak treadmill speed (3), have been used to measure the performance of long-distance athletes with different training status. ${\dot{\text{V}}\text{O}}_{2\max}$is widely accepted as the upper limit of a body's aerobic functioning (1), which is considered a key component of endurance capacity (4). In turn, running economy, metabolic thresholds, and peak treadmill speed are related to energetic efficiency (5), cellular acidosis (6), and anaerobic metabolism (7), respectively. Therefore, the relationship between these physiological variables and endurance performance appears to owe to the ability to remove metabolites related to fatigue, as well as resynthesizing ATP aerobically and anaerobically. In addition, it was suggested that the distribution of exercise intensity in a race [i.e., pacing) is also a relevant factor for athletic performance (8). Thus, the distribution of exercise intensity can be determined by monitoring physiological responses to avoid premature fatigue and maximize performance (9).

Recent studies have demonstrated that running intensity varies considerably during middle and long-distance events (8) and is associated with the interplay between energy systems. Hettinga et al. (10) found that aerobic metabolism increased until the end of a 4-km cycling time trial, whereas anaerobic metabolism was greatest during the last part of the race. In addition, Nummela et al. (11) found a positive association between the speed of the final lap in a 5-km running time trial and the highest speed measured in a maximal anaerobic running test. Collectively, these findings suggest that the interaction between aerobic and anaerobic metabolism may be decisive in these kinds of events.

The contribution of both metabolisms is important, and an estimation of the contribution of energy systems during exercise can be determined by the energy equivalent of O_2_ (12,13). Previous studies have used oxygen uptake and oxygen energy equivalents derived by blood lactate accumulation to estimate the contribution of aerobic and glycolytic metabolisms, respectively (12,14,15). More recently, Bertuzzi et al. (13) used this method to analyze the profile of energy contribution by aerobic and glycolytic systems during an incremental exercise test (IET). These authors demonstrated that the relative aerobic contribution progressively decreased by only 9% from the first to last stage, suggesting this pathway was predominant throughout the IET. In addition, it was observed that glycolytic metabolism did not contribute largely to the energy expenditure at intensities above the anaerobic threshold. Because the contribution of the energy systems is important for determining variations of exercise intensity and overall performance, it would be interesting to verify whether the energy contribution during an IET could be related to running speed in different sections of an endurance event. This might help coaches and sports scientists to develop specific training programs and understanding pacing strategies for specific events.

Therefore, the purpose of this study was to investigate the relationship between the relative contributions of the aerobic and glycolytic systems, as well as the performance and running speed in different sections of a 10-km running time trial. Based on past studies showing that aerobic metabolism is predominant in events with different distances (16), and increases from the start until the end of cycling time trials (10), whereas anaerobic metabolism is important in the middle and final sections of time trials (10,17), we hypothesized that energy system contribution is correlated to overall performance, aerobic energy system contribution is correlated with speed at different sections of a time trial (start, middle, and end phase) and that glycolytic energy system contribution is strongly correlated with the end phase of a race. Therefore, this would represent an alternative method for assessing endurance runners.

## Material and Methods

### Participants

Thirteen male, recreationally trained long-distance runners (means±SD: age: 32.3±7.5 years; weight: 68.2±10.2 kg; height: 173.1±10.9 cm) volunteered to participate in this study. All participants regularly competed in 10-km running races at a regional level. They also had personal records of 40±5 min in 10-km running races, had performed at least ten 10-km running races in the last 2 years, and had trained in the last 3 years without interruption. The subjects' running training volume was 38.6±4.4 km/week, reported as the mean distances covered, assessed through a training log recorded for 2 weeks prior to the beginning of the study and for the following 2 weeks. The participants received a verbal explanation about the possible benefits, risks, and discomfort associated with the study, and signed a written informed consent before study participation. All subjects were healthy non-smokers with no cardiovascular or neuromuscular diseases. The study was previously approved by the Ethics Committee for Human Studies of the Escola de Educação Física e Esporte, Universidade de São Paulo.

### Experimental design

Each subject visited the university on two separate occasions within a 7-day period, with each session separated by at least 48 h. Each completed: a) an IET to exhaustion on a treadmill for the determination of the respiratory compensation point (RCP), ${\dot{\text{V}}\text{O}}_{2\max}$, peak treadmill speed (PTS) and energy system contributions; and b) a 10-km time trial (T10-km) on an outdoor track to determine overall endurance performance and pacing. The subjects were instructed to refrain from exhaustive or unaccustomed exercises and the ingestion of caffeinated or alcoholic beverages for 24 h before the tests.

### Procedures

*Incremental exercise test*. Before starting the IET, participants were asked to rest quietly in a standing position for 5 min to determine the baseline ${\dot{\text{V}}\text{O}}_{2}$(${\dot{\text{V}}\text{O}}_{2}$baseline). After a 5-min warm-up at 12 km/h, the test was started with a treadmill speed of 13 km/h; then the speed was increased by 1 km/h every 3 min until volitional exhaustion. The treadmill (model TK35, CEFISE, Brazil) was set at a gradient of 1% throughout the IET to simulate outdoor running (18). Subjects received strong verbal encouragement to continue as long as possible. Each stage was separated by a 10-s rest period, during which capillary blood samples (25 µL) were obtained from the earlobe and analyzed for blood lactate concentrations (YSI 1500; Yellow Springs Instruments, USA). Breath-by-breath pulmonary gas exchange data were collected continuously using a gas analyzer (Metalyzer 3b, Cortex, Germany) and a mean value was determined over consecutive 20-s periods. Calibration of the device was performed according to manufacturer specifications using ambient air of known composition containing 20.9% O_2_ and 5% CO_2_, and a 3 L syringe (Model 5530 Series, Hans Rudolph, USA). Heart rate was measured during the test with a heart rate monitor coupled to the gas analyzer (S810, Polar Electro Oy, Finland). The maximal heart rate was defined as the highest value obtained at the end of the test. The ${\dot{\text{V}}\text{O}}_{2\max}$was determined when two or more of the following criteria were met: an increase in ${\dot{\text{V}}\text{O}}_{2}$of less than 2.1 ml/kg/min on two consecutive final stages, a respiratory exchange ratio greater than 1.1, or a heart rate±10 bpm of the maximal age-predicted heart rate (19). The RCP was determined by three independent investigators as the point of a nonlinear increase in the $\dot{\text{V}}\text{E}/{\dot{\text{V}}\text{CO}}_{2}$, a constant increase in the $\dot{\text{V}}\text{E}/{\dot{\text{V}}\text{O}}_{2}$, and the first decrease in the expiratory fraction of CO_2_ (20). The highest velocity achieved during the test was recorded as the PTS. When subjects were not able to complete the last stage, the PTS was calculated from the following equation (21): PTS=LCS + (TLIS/180 × speed increment) where LCS is the velocity in the last complete stage performed by the subject, and TLIS is the time in seconds sustained by the subject in the last incomplete stage.

*10-kilometer running time trial*. Participants performed the T10-km on an outdoor 400-m track and were instructed to finish the race as quickly as possible, as if in a competitive event. Before the trial, the participants warmed up for 10 min at 12 km/h. They were instructed to maintain regular water consumption within 6 h of testing and water was provided *ad libitum* during the entire event. Verbal encouragement was provided during the entire event. Speed and heart rate were registered at each 100 m via a global positioning system (GPS Forerunner¯ 405, Garmin, USA) and the mean velocity of each section was calculated. Maximal heart rate was the highest value obtained at the end of the test. Ambient temperature and humidity values were provided by the Institute of Astronomy, Geophysics and Atmospheric Sciences of the University of São Paulo, São Paulo, SP, Brazil. The means±SD values for temperature and humidity were 22.8±1.3°C and 61.0±4.2%, respectively.

*Calculation of energy system contributions.* Energy system contributions during each stage of the IET were determined as previously described (22,23). Briefly, the net aerobic energy system was estimated by subtracting ${\dot{\text{V}}\text{O}}_{2}$baseline from the ${\dot{\text{V}}\text{O}}_{2}$exercise area integrated over time by the trapezoidal method. To estimate the glycolytic system contribution, a value of 1 mM [La-]net was considered to be equivalent to 3 mL O_2_/kg body mass (24). Because the ATP-PCr system is predominantly used to resynthesize of ATP during high-intensity (3-5 times the power output that elicits ${\dot{\text{V}}\text{O}}_{2\max}$), short duration exercises (∼10 s) (25), and the contribution of alactic metabolism was not considered. The aerobic (W_AER_) and glycolytic (W_GLYCOL_) energy system contributions represented the sum of all stages in IET. The total metabolic demand (W_TOTAL_) was the sum of both energy metabolism contributions. W_AER_ and W_GLYCOL_ are also reported as a percentage of W_TOTAL_. A caloric equivalent of 20.9 kJ/L O_2_ was used for the energy systems calculations.

### Statistical analyses

The distribution of the data was analyzed using the Shapiro-Wilk test, and the results showed a normal Gaussian distribution. Data are reported as means±SD. Paired *t*-tests were used to compare the W_AER_ and W_GLYCOL_ contributions of each stage during the IET. Pearson's correlation coefficient was calculated to assess the relationship between the energy system contributions and physiological variables (PTS, RCP, and ${\dot{\text{V}}\text{O}}_{2\max}$) with partial running speed sections (start: first 400 m, middle: from 400 to 9600 m, and finish: last 400 m) and overall performance. The statistical power for the correlations was calculated as 1-β, and is reported as a percentage (%). The level of significance was set at α=0.05. All statistical analyses were conducted using the SPSS statistical package (version 16.0, USA).

## Results

### Energy system contributions during the incremental exercise test

Table 1 shows the physiological and performance parameters measured during the IET, and the heart rate and section running speeds during the T10-km. Table 2 shows the energy system contributions reported as absolute and percentage values for each stage of the IET. The W_AER_ was significantly higher compared with W_GLYCOL_ throughout the IET (P<0.05). The W_AER_ increased continuously up to 223.3±30.0 kJ, while W_GLYCOL_ increased continuously up to 45.8±8.8 kJ. When reported as a percentage, the W_AER_ continuously decreased from 94.6±10.2% at 13 km/h to 82.9±11.0% at 18 km/h, while the W_GLYCOL_ continuously increased from 5.4±3.5 to 17.1±3.3%.

**Table:**
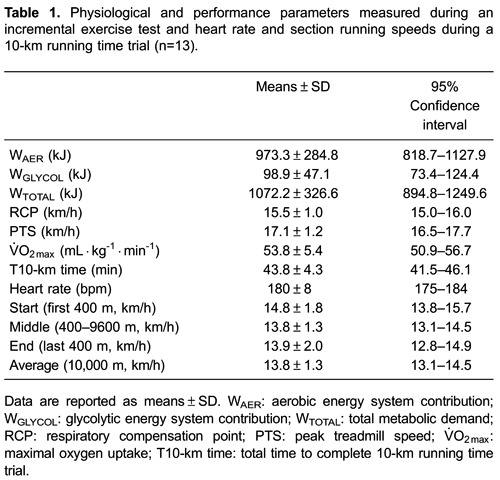

**Table:**
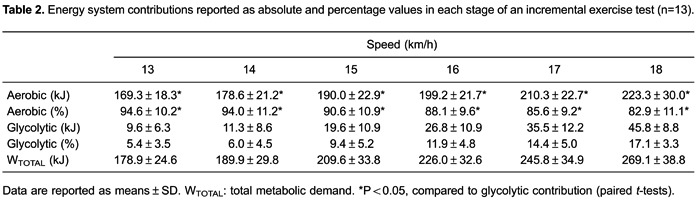

### Ten-kilometer running performance

The W_AER_ (r=-0.85; P=0.01), W_GLYCOL_ (r=-0.67; P=0.01), and W_TOTAL_(r=-0.83; P=0.01) estimated from the IET were significantly correlated with the T10-km time. The PTS (r=-0.78; P=0.01), RCP (r=-0.56; P=0.04), and ${\dot{\text{V}}\text{O}}_{2\max}$(r=-0.67; P=0.01) were also significantly correlated with the T10-km time. Table 3 shows the Pearson's correlation coefficient of the running speed in different sections of the T10-km with the energy system contribution and physiological variables. The W_AER_, W_GLYCOL_, and W_TOTAL_ were positively and significantly correlated with the running speed in the middle and final sections and with the mean speed in the T10-km. Similarly, PTS also correlated with the middle and final sections, and mean speed in the T10-km, while RCP correlated with the middle section and mean speed. However, ${\dot{\text{V}}\text{O}}_{2\max}$did not correlate with any of the speed sections and none of the variables correlated with the start section (Table 3).

**Table:**
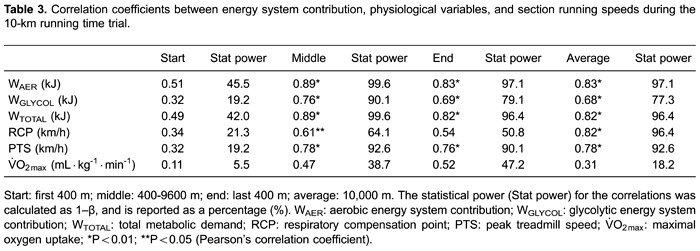

## Discussion

The present study was conducted to determine the association between performance in a T10-km and energy system contributions estimated during an IET. Our results showed that W_AER_, W_GLYCOL_ and W_TOTAL_ were significantly correlated with the T10-km time. Additionally, W_AER_, W_GLYCOL_, and W_TOTAL_ were positively and significantly correlated with speed in the middle and final sections of the T10-km. These findings indicated that an estimation of aerobic and glycolytic metabolism during IET is an alternative variable to be considered for the evaluation of long-distance runners, with the additional advantage of being related to specific sections of a T10-km run.

Because the intensity of exercise progressively increases throughout the IET, the contribution of energy systems is altered to meet the increasing energy demand. Although most sport scientists and coaches believe that metabolic pathways have a transitional nature during the IET, only one study has quantified the profiles of aerobic and glycolytic metabolism during an IET (13). It was demonstrated that the aerobic contribution was predominant during all stages of the IET and that there was an increase in the glycolytic contribution at intensities equal to or above the anaerobic threshold. These results are in accordance with the data of the current study, because the W_AER_was significantly higher than W_GLYCOL_ throughout the IET. Therefore, the results of the current study confirm the idea that aerobic metabolism is predominant throughout IET and that the glycolytic contribution becomes substantial, but not predominant, during the last stages of an IET (13).

Our results also showed that the correlation coefficient between T10-km time and W_TOTAL_ was higher than between the T10-km time and the other variables traditionally related to endurance performance (i.e., PTS, RCP, and ${\dot{\text{V}}\text{O}}_{2\max}$). In addition, the W_AER_ and W_GLYCOL_ were also significantly correlated with the overall running performance during the T10-km. These correlations observed between energy system contributions during IET and T10-km might be associated with the energy profile of those undertaking long-distance events. Previous findings indicated a high relative aerobic contribution (96%) during long-distance running, suggesting the importance of oxidative metabolism for overall running performance (26). Therefore, aerobic contribution might be related to endurance performance because of the high capacity (total amount of available energy) of aerobic metabolism to resynthesize ATP, so it can be recruited at high magnitudes over a long period of time (25). Furthermore, Duffield et al. (16) demonstrated that aerobic metabolism provided the major pathway for energy supply to runners of 1500- and 3000-m track events, with the aerobic contribution increasing as the event distance increases. In turn, the high rate of energy production (power) of anaerobic metabolism can influence endurance performance and determine the ability of an athlete to accelerate in specific sections of a long distance race, for example a sprint at the end (27). Furthermore, Lazzer et al. (28) determined the effects of long-lasting endurance events on the energy cost of running and showed that a substantial increase in the cost of running during the competition caused a marked worsening of performance. Therefore, athletes with a high ability to resynthesize ATP aerobic and anaerobically during IET might also be able to maintain higher speeds during a running race.

In the current study, the contributions of aerobic and glycolytic metabolism determined during the IET were also significantly correlated with the middle and final sections of the T10-km. These findings are in agreement with previous studies showing changes in both aerobic and glycolytic metabolism in events performed with different pacing strategies (10,17,29). Hettinga et al. (10) demonstrated that in 4000 m cycling time trials, aerobic contribution increased toward the end of the race, independent of strategy, but the power distribution during these races appeared to be regulated primarily by changes in anaerobic contribution. Santos et al. (29) reported that the time to complete a 4-km cycling time trial was negatively associated with the total anaerobic work expended (r=-0.77). Thus, it seems that because of the relationship with the aerobic and anaerobic contribution during a race, estimating the energy system contributions during IET to evaluate the endurance of athletes has an additional advantage in predicting the performance during specific sessions (i.e., middle and end phases) of long-distance events.

In relation to physiological variables, the middle section of the T10-km was correlated with PTS and RCP, while the final section was correlated with PTS only. Considering that the intensity of the end sprint seems to be dependent on anaerobic energy expenditure (10), the correlation of PTS with the final section could be expected, because it is also influenced by anaerobic characteristics (30). In turn, a possible advantage in attaining the RCP at higher intensities could be the achievement of higher speeds without the accumulation of metabolites related to muscle fatigue. RCP occurs in response to an initial decrease of blood pH, which represents the beginning of failure of muscle buffering capacity (31). Foster et al. (32) suggested that athletes learn how to monitor values of muscle pH and adjust their pacing accordingly so that they ideally reach critically low values of pH near the end of a race. This could preserve the capacity of skeletal muscle to produce force during the middle section. Because the last part of a race might not be long enough for the establishment of acidosis, athletes could attain high speeds independently of muscle buffering capacity. This might explain the lack of correlation between RCP and running intensity during the final section.

In turn, ${\dot{\text{V}}\text{O}}_{2\max}$was not correlated with any speed sections in the T10-km. This result is in contrast with previous findings (33), but similar with results of another study conducted by Lima-Silva et al. (34) in which the athletes covered a similar distance during a running trial. Although ${\dot{\text{V}}\text{O}}_{2\max}$is considered an important determinant of elite performance in competitive distance running (1), it may not be sufficiently sensitive to predict performance in specific sessions of a T10-km run. In particular, previous findings suggested that ${\dot{\text{V}}\text{O}}_{2\max}$might not be a good predictor of endurance performance in homogeneous groups (3). Despite the absence of studies including specific running speed sections, several studies have shown that other variables (i.e., PTS) besides ${\dot{\text{V}}\text{O}}_{2\max}$were related to endurance performance (3,35). Thus, at least for running speed sections, ${\dot{\text{V}}\text{O}}_{2\max}$might not be a good predictor of running pace in a T10-km.

It was interesting to observe that no correlation was obtained with the first 400 m of the T10-km. It was previously demonstrated that the time needed for the stabilization of physiological variables (e.g., oxygen uptake) that are important for this event was greater than the time required to complete this first section of the run (36). In this context, Bertuzzi et al. (33) demonstrated that in a 10-km running time trial, only the rate of perceived exertion accounted for the variance of speed during the start phase. Therefore, the authors suggest that psychological factors may be more important during the early stages of a running race than physiological factors (33). This could explain the results found in the present study, because the first 400 m were not correlated with any measured physiological variable.

In conclusion, the results of the present study demonstrated a positive and significant correlation between W_AER_, W_GLYCOL_, and W_TOTAL_ with the running speed in the middle and final sections of a 10-km running time trial. These findings showed that the assessment of energy system contributions during IET may be potentially useful as an alternative variable to evaluate endurance runners, and assess a runner’s performance in specific parts of a long distance race. From a practical standpoint, sports scientists and coaches could use these variables measured during an IET, particularly W_TOTAL_, as an alternative method to diagnose the training status of endurance athletes. This might be especially relevant because the application of knowledge regarding energy system contributions involved in any athletic event is considered important for the correct administration and structuring of training regimes to achieve peak athletic performance (16).
